# A face recognition algorithm based on the combine of image feature compensation and improved PSO

**DOI:** 10.1038/s41598-023-39607-3

**Published:** 2023-07-31

**Authors:** Yan Lijuan, Zhang Yanhu

**Affiliations:** Guangdong Songshan Polytechnic, Shaoguan, 512126 Guangdong China

**Keywords:** Developmental biology, Evolution

## Abstract

Face recognition systems have been widely applied in various scenarios in people's daily lives. The recognition rate and speed of face recognition systems have always been the two key technical factors that researchers focus on. Many excellent recognition algorithms achieve high recognition rates or good recognition speeds. However, more research is needed to develop algorithms that can effectively balance these two indicators. In this study, we introduce an improved particle swarm optimization algorithm into a face recognition algorithm based on image feature compensation techniques. This allows the system to achieve high recognition rates while simultaneously enhancing the recognition efficiency, aiming to strike a balance between the two aspects. This approach provides a new perspective for the application of image feature compensation techniques in face recognition systems. It helps achieve a broader range of applications for face recognition technology by reducing the recognition speed as much as possible while maintaining a satisfactory recognition rate. Ultimately, this leads to an improved user experience.

## Introduction

Recently, non-contact and non-cooperative face recognition technology has become increasingly popular. Many researchers have been studying this field^1–7^. Face recognition can be divided into three categories: global image-based, deep neural networks based and local feature-based recognition.

One of the most famous global image-based recognition approaches is Eigenfaces^8^. It first uses principal component analysis (PCA) to reduce the dimensionality of the original image, and then conducts training and recognition. Kim et al.^9^ proposed another method called kernel Principal Component Analysis (KPCA), which is based on kernel nonlinear local feature description and PCA method. However, these methods have poor robustness in scenarios where facial expressions and environments change frequently. In order to address this problem, Lu et al.^10^ proposed a method based on Kernel discriminant analysis to improve the robustness of the face recognition system. This method can ignore facial expression and environment. Experimental results showed that this method is better than kernel Principal Component Analysis (KPCA) and Generalized Discriminant Analysis (GDA). Weber Local Descriptor (WLD)^11^ is popular with its simplicity and efficiency, and many scholars devoted themselves to the research in this field. However, the original method has an insufficient of sensitive to lighting conditions. In order to solve this problem, lots of scholars devoted in to improving its robustness to lighting conditions^12–16^. Among them, an improved weber local circle gradient pattern (WLCGP) algorithm covering a wider surrounding area in addition to the values of eight locations around the pixels has been introduced by Fang et al.^17^. The WLCGP algorithm designed by Fang et al. can extract feature information with a wider screening range and can better deal with the problem of picture lighting sensitivity. Unfortunately, due to the huge calculations relatedly to the method, the processing speed of the WLCGP algorithm is slow.

Lots of other researches focused on global image implementation. Deng et al.^18^ proposes a new loss function, called the additive angular margin loss, for improving the performance of deep face recognition. The loss function introduces an angular margin to the softmax loss, which enhances the discrimination power of the features. The proposed method achieves state-of-the-art results on several face recognition benchmarks. Liu et al.^19^ proposes a novel attention mechanism, called channel attention, for improving the performance of residual networks in face recognition. The mechanism is integrated into the residual blocks of the network, and it selectively amplifies the informative channels while suppressing the non-informative ones. The proposed method achieves state-of-the-art results on several face recognition benchmarks. Zhang et al.^20^ proposes a multi task learning framework for jointly performing face detection, alignment, and recognition using a single deep neural network. The framework consists of multiple stages, and each stage performs a specific task. The proposed method achieves state-of-the-art results on several face recognition benchmarks. Liu et al.^21^ presents a large-scale face dataset that contains over 200,000 celebrity images with 40 attribute annotations per image. The dataset is designed to facilitate research in face analysis, including face recognition, attribute prediction, and landmark detection. Schroff et al.^22^ proposes a unified embedding framework for face recognition and clustering. The framework employs a siamese neural network to learn a feature representation that maps similar faces to nearby points in the embedding space and dissimilar faces to distant points. The proposed method achieves state-of-the-art results on several face recognition benchmarks.

Since deep neural networks (DNN) has been introduced in face recognition field, many scholars have done lots of work to apply the technology into face recognition system. Lots of them have got outstanding achievement, and do well in face recognition accuracy. Zhang et al.^23^ have proposed a multi task cascaded convolutional network (MTCNN) that can perform joint face detection and alignment using a single model. The MTCNN comprises three stages, where the first stage detects faces, the second stage refines the bounding boxes, and the third stage aligns the faces. This network has shown state-of-the-art performance on various face detection and alignment benchmarks. Papyan et al.^24^ have presented a method for combining face and ear biometrics to identify individuals in uncontrolled environments. The proposed method extracts unique features from both the face and ear images and then merges them using a deep neural network. Several public datasets were used to evaluate this approach, and the results suggest that combining face and ear biometrics can significantly enhance the performance of person identification in unconstrained environments. Shi et al.^25^ proposed a real-time face alignment method called the Coarse-to-Fine Auto Encoder Network (CFAN). The CFAN has three stages that gradually refine face landmarks from coarse to fine. This method was trained on a large dataset and achieved state-of-the-art performance on several face alignment benchmarks. Gong et al.^26^ presented a pose-aware model that achieves pose-invariant face recognition in the wild. This model was trained on a large-scale dataset and used a combination of convolutional neural networks (CNNs) and pose regression networks. The proposed method outperforms state-of-the-art methods on several face recognition benchmarks. Zhu et al.^27^ proposed a 3D solution for face alignment across large poses. This method used a 3D face model to generate synthetic faces corresponding to different poses and trained a CNN to align faces across large poses. The proposed method achieved state-of-the-art performance on several face alignment benchmarks. Additionally, there are many other papers on deep learning in the field of face recognition research, including^28–30^.

For the local feature-based face recognition approaches, there are also various research results. The Histogram of Oriented Gradients (HOG) algorithm, which is widely used in face recognition and target detection, is one of the classical methods, but is sensitive to changes in image size and rotation. To overcome this, Lowe proposed the Scale-Invariant Feature Transform (SIFT) algorithm^31^, which performs robustly to changes in image size and rotation. However, the computational cost of the SIFT algorithm increases exponentially with the number of extracted feature points, limiting its applicability. Local Binary Pattern (LBP)^32^ is another popular face recognition algorithm based on local features and is widely used in computer image recognition. LBP algorithms and their extensions^33–36^ play a critical role in this field. Chen et al.^33^ proposed a novel image feature description by combining the LBP method with the Shearlet-decomposition method, which effectively reduces image noise and performs well. Ahonen et al.^34^ introduced a native LBP method that is robust to varying image illuminations. Jun and Kim developed the Local Gradient Pattern (LGP)^35^ algorithm, which can capture the intensity distribution of the gradient and achieve local variation near key points. This method was initially developed for feature analysis and to address incomplete feature extraction in the LBP algorithm for extracting facial features. Wang et al.^36^ proposed the Complete Local Binary Mode (CLBP) face recognition algorithm based on the traditional LBP algorithm. They also introduced a classification recognition method based on local difference and central pixel gray value analysis on the basis of LBP.

There are also many other methods which have been well researched for face recognition based on local feature-based recognition. Qin et al.^37^ surveyed recent advances in deformable face recognition, which is an emerging direction in face recognition research. Deformable face recognition aims to address the challenges caused by non-rigid face variations, such as expression, pose, and aging. The paper provides a comprehensive review of deformable face recognition methods, including feature representation, metric learning, and model architecture. Wang et.al proposed^38^ a novel unsupervised domain-specific data augmentation technique for multi-modal face recognition, which utilizes data from multiple sources to improve the recognition performance. The proposed method generates realistic synthetic data by learning the data distribution of each modality in a domain-specific manner, and then synthesizing new samples using generative models. The experimental results demonstrate the effectiveness of the proposed method on several benchmark datasets. Zhang et al.^39^ proposes a domain adaptive meta-learning framework for face recognition, which aims to address the domain gap problem caused by variations in illumination, expression, and pose. The proposed method learns a domain-invariant feature representation through meta-learning, and then adaptively adjusts the feature representation for each domain through domain-specific calibration. The experimental results demonstrate the superior performance of the proposed method on several benchmark datasets. Amirhossein et al.^40^ proposed a novel method for face recognition that utilizes the geometry of the feature space to improve the discriminative power of the feature representation. The proposed method learns a low-dimensional embedding of the feature space, which preserves the pairwise distances between the features. The experimental results demonstrate the effectiveness of the proposed method on several benchmark datasets. Zhang et al.^41^ proposes a novel approach for face recognition that utilizes the temporal dynamics of facial regions to improve the recognition performance. The proposed method extracts the features of different facial regions over time, and then models the temporal dynamics of these features using a recurrent neural network. The experimental results demonstrate the superior performance of the proposed method on several benchmark datasets.

Most of the face recognition algorithms above involve the setting of parameters, and the values of the parameters directly affect the recognition rate of the face recognition system. Lots of aforementioned literatures directly give the relevant parameters which have been tested when set the relevant parameters.

The setting of parameter values has a significant impact on the facial recognition system, and optimizing the feature compensation coefficients involves a large amount of work. The efficiency of manual optimization methods is low. Finding the optimal combination of feature compensation coefficients while improving work efficiency is a challenging problem that urgently needs to be addressed in this research. In view of this situation, incorporating intelligent algorithms into the facial recognition system can effectively solve this problem.

Among numerous intelligent algorithms, the Particle Swarm Optimization (PSO) algorithm has excellent collective collaboration ability. This characteristic enables it to obtain the optimal solution in a shorter time. Therefore, in this study, the PSO intelligent algorithm is selected to solve the problem of finding the optimal solution for feature compensation coefficients.

Incorporating intelligent algorithms has been an approach to enhance the adaptability of face recognition systems. Shin et al.^42^ introduced an elastic graph matching and identification features analysis algorithm that considers image position changes and proposes a cost function. A clustering algorithm is used to optimize the function in their work. Krisshna et al.^43^ proposed a feature description selection algorithm that uses the PSO algorithm. Their approach integrates DWT, DFT, and DCT operators and utilizes the ThBPSO algorithm to choose the best operator in the transformed feature graph. Mistry et al.^44^ suggested an MGA-embedded PSO algorithm that combines PSO and GA methods for optimizing image feature descriptions. Simulation experiments showed that the proposed approach outperformed traditional PSO, simultaneous PSO transformation algorithm, classical GA algorithm, and other related face recognition algorithms. Other scholars have also conducted similar optimization studies^45–48^.

The methods mentioned above aim to improve the face recognition algorithm by selecting the best feature description from multiple options. Building on these ideas, Preethi et al.^49^ proposed an improved PSO algorithm that selects multiple feature descriptions simultaneously to enhance the face recognition system's ability to handle changes in image features. Experimental results show that this method can significantly increase the accuracy of the face recognition system without adding computational complexity through complex transformations. However, this algorithm is not very robust to changes in illumination and expression because it searches for multiple feature factors to describe the image features. Ahmed et al.^50^ proposed a method that combines the Gabor wavelet transform for feature extraction and the PSO algorithm for optimizing the image feature description, followed by a 6-layer deep learning method for face recognition. Experimental results on the ORL and YALE face datasets demonstrate that this approach effectively improves the face recognition rate.

However, in this method, the PSO algorithm is used directly to optimize the image feature description, rather than optimizing the coefficient coupling relationship of multiple feature descriptions. Zhang et al.^51^ proposed an Image Gradient Feature Compensation (IGFC) algorithm for face image recognition based on local feature compensation. However, the feature compensation coefficients of image feature descriptions need to be optimized, which can be a challenging task. In the manual approach used in^51^, more suitable feature coefficients cannot be obtained efficiently due to the exponential increase in the number of tests when the number of calculation factors for image feature compensation increases. This poses a challenge in setting appropriate feature compensation coefficients for different datasets.

Other studies have explored the use of particle swarm optimization (PSO) and image feature compensation techniques to improve face recognition technology. Zhang et al.^52^ conducted a similar study, but there are several differences between their approach and the one in this study. Firstly, this study uses a more flexible compensation strategy that combines addition and subtraction based on the compensation sequence. Secondly, the pixel values of the compensation factor S7 are optimized based on the number of non-zero pixel values around each pixel, which results in more delicate pixel extraction. Thirdly, a new strategy for reconstructing the pixel value calculation is introduced when constructing the compensation factor S8, which allows for better feature extraction. If there are less than three non-zero values around a pixel, it is set to zero. Otherwise, the pixel value is assigned based on its position in the image, using either the average value of the surrounding pixels or the nearby four-pixel averaging method.

The paper proposes a new fusion compensation and improved PSO algorithm (FCAI). The proposed algorithm aims to optimize the feature compensation coefficients in the face recognition process.

The paper makes four main contributions: (1) improving the image feature compensation technique, (2) designing commonly used image feature compensation calculation factors, (3) proposing a novel method to extract feature description of the compensated original image, and (4) using an improved PSO algorithm to solve the optimal combination of feature compensation coefficients when there are multiple computational factors.

The paper is structured as follows: Section “Relevant concepts” explains related concepts, Section “Recognition algorithms” presents the framework model of the face recognition algorithm, Section “Compensation coefficient solving algorithm” designs the improved PSO algorithm, Section “Experiments” verifies the algorithm through experiments, and Section “Conclusions” concludes the paper.

## Relevant concepts

### Feature compensation

This paper proposes a new method for improving face recognition inspired by^51^. The method is based on a statistical gray value strategy that uses principal component analysis to enhance the recognizability of images and improve the system's recognition rate. To implement the proposed method, the original image A is transformed to obtain a main feature information description matrix that includes computational factors S1, S2, S3…Sn. Each computational factor has a compensation coefficient (f1, f2, f3… fn) that adjusts its contribution to the feature extraction of image A. The proposed method compensates for the features of the original image and then extracts the image feature description to enhance the image recognizability and improve the recognition rate of the system.

The main purpose of image feature compensation is to enhance specific characteristics of an image, weaken common features, and improve the image's recognizability. The computational factors are extracted from the original image through various transformations and contain image feature information that can be enhanced or attenuated.

The feature graph Sig is calculated using Eq. (1), which is designed to better represent the feature information of image A. The conversion process is intended to adjust the pixel value distribution of the original image and enhance its feature information. This method is based on previous research and aims to boost the system's recognition accuracy. Equation (1) calculates Sig as A plus a series of weighted feature compensation coefficients, where each weight (Si) is a calculation factor for image A and each feature compensation coefficient (fi) is multiplied by Si.

1 $$Sig \, = \, A \, + \, \left( { - 1} \right)^{1} *S_{1} * \, f_{1} + \, \left( { - 1} \right)^{2} *S_{2} * \, f_{2} \ldots .. \, + \, \left( { - 1} \right)^{n} *S_{n} *f_{n}$$

The paper compared the histograms of the original image and the image after feature compensation processing to visually and intuitively compare the feature information of the two. Feature compensation processing changes the layout of the compensated image histogram and generates new pixel values. The improvement in image recognition after feature compensation depends on the calculation factor and the feature compensation coefficient.

The study used 8 computational factors to compensate the image and tested a range of feature compensation coefficients from – 10.00 to 10.00 with an accuracy increase of 0.01. There were 2000 types of compensation methods using one computational factor, and the combination of 8 computational factors for combined compensation is a very large value that cannot be completed manually.

The process of feature compensation requires the use of feature compensation calculation factors. Several common feature compensation calculation factors are listed in Section “Calculation factors proposed”.

### Calculation factors proposed

This paper proposes several methods for calculating image feature compensation factors in order to implement the feature compensation strategy for the original image.

The contents of factors 1 to 6 refer to reference^51^.

#### Factor 1: left offset matrix

The first method is the left offset matrix, which is obtained by subtracting the pixel value from its corresponding value of the left pixel in the grayscale map and taking the absolute value. This method utilizes the fact that the pixel values of the same color points in the grayscale map are identical, and it provides contour information for the face image. The calculation factor for the left offset matrix is denoted as S1, and its calculation process is shown in Eqs. (2) and (3).

2 $${S}_{t1}(i,j)=\left\{\begin{array}{l}A\left(i+1,j\right) i\ge 1 \; and \; i<m\\ {S}_{t1}\left(i-1,j\right) i=m\end{array}\right.$$

3 $$S1 = \left| {A - St1} \right|$$

Other Factors are defined as:

#### Factor 2: right offset matrix

The calculation process of S2 is shown in Eqs. (4) and (5).

4 $${S}_{t2}(i,j)=\left\{\begin{array}{l}A\left(i-1,j\right) i>1 and i\le m\\ {S}_{t2}\left(i+1,j\right) i=1\end{array}\right.$$

5 $$S2 = \left| {A - St2} \right|$$

#### Factor 3: upper offset matrix

The calculation process of S3 is demonstrated in Eqs. (6) and (7).

6 $${S}_{t3}(i,j)=\left\{\begin{array}{l}A\left(i,j-1\right) j>1 and j\le n\\ {S}_{t3}\left(i,j+1\right) j=1\end{array}\right.$$

7 $$S3 = \left| {A - St3} \right|$$

#### Factor 4: lower offset matrix

The calculation process of S4 is shown in Eqs. (8) and (9).

8 $${S}_{t4}(i,j)=\left\{\begin{array}{l}A\left(i,j+1\right) j\ge 1 and j<n\\ {S}_{t4}\left(i,j-1\right) j=n\end{array}\right.$$

9 $$S4 = \left| {A - St4} \right|$$

#### Factor 5, 6 are given as

10 $$S5 \, = \, \left( {S1 \, - \, S2} \right) \, + \, \left( {S3 \, - \, S4} \right)$$

11 $$S6 \, = \, S1 \, + \, S2 \, + \, S3 \, + \, S4$$

#### Factor 7: Feature map noise reduction

S6 is a calculation factor that contains the majority of the contour information of the original image. Nevertheless, S6 also includes a substantial amount of non-major contour information. To extract the most essential feature information of the original image, the paper carried out an additional noise reduction process on S6. The aim of this process was to eliminate the core contour information of S6.

The denoising process of the calculated factor S6 is defined as follows:

1. Find the value *aveV* of image S6, and its calculation equation is defined as:
  12 $$aveV= Min(Median\left(S\left(i,j\right)\cong 0\right),Average\left(S\left(i,j\right)\cong 0\right)) i\ge 1 and i<n and j\ge 1 and j<m$$
  where *Median(S(i,j))* is the median value of S6, and *Average(S(i,j)* is the average value of S6.
2. All pixels whose pixel value is less than aveV* fx in image S6 are set to 0 to obtain the new feature description S7 of the original image, and the implementation process is indicated in Eq. (13).
  13 $${S}_{7}(i,j) =\left\{\begin{array}{c}0 S\left(i,j\right)<aveV*fx*\frac{count(S\left(i,j\right)\cong 0)}{count(S\left(i,j\right)=0)}\\ S\left(i,j\right) S\left(i,j\right)>aveV*fx* \frac{count(S\left(i,j\right)\cong 0)}{count(S\left(i,j\right)=0)}\end{array}\right.$$

The formula for calculating the new factor S7 involves variables such as count(S(i,j) ≅ 0), count(S(i,j) = 0), and fx. These variables determine how much core contour information is retained in S6. After applying a noise reduction process to S6, the resulting S7 is the new calculation factor that contains the most essential feature information of the original image.

#### Factor 8: feature amplification

A new calculation factor S8 is generated based on S7 to improve the proportion of core contour information in it. An algorithm is designed to expand the influence range of the core contour information in the implementation process of S8. The process involves the following steps:

Counting the effective pixel values in each 9-grid of S7, where an effective pixel value is defined as a pixel with a value greater than 0.

Calculating the mean Vave of all non-zero pixel values in the 9-grid.

**Figure 1:**
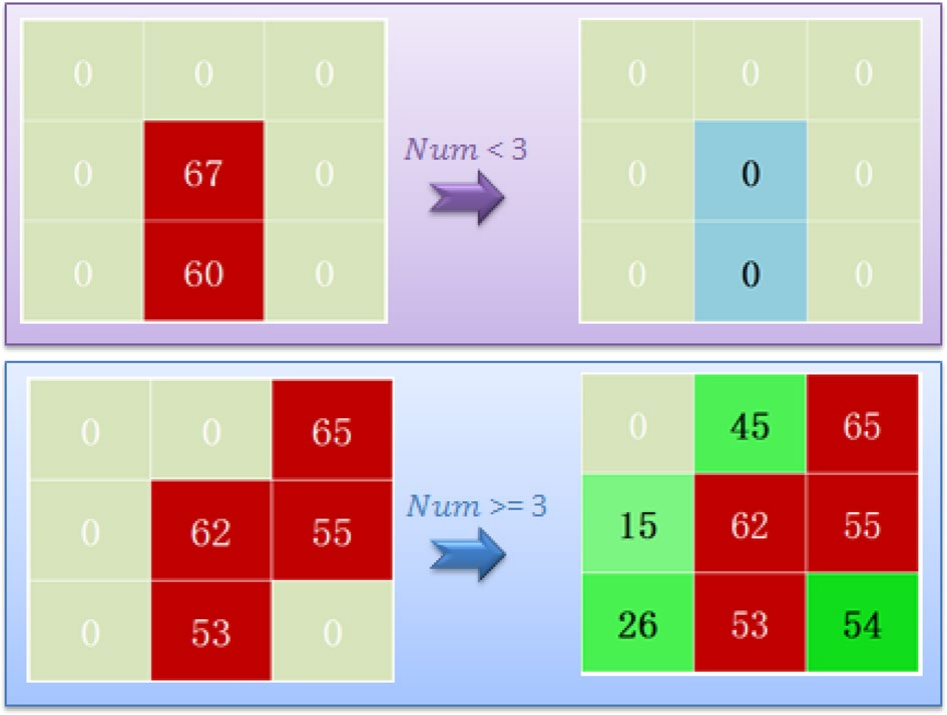
Image feature magnification effect demonstration.

Setting the pixel values of all pixel points in a certain 9-grid with a value of 0 to a value determined by Eq. (16) if the number of valid pixels in that 9-grid is greater than or equal to 3. If the number of valid pixels in the 9-grid is less than 3, all values in the 9-grid are set to 0. The process is illustrated in Fig. 1.

Its realization process can be seen in Eqs. 14–16.

14 $$Num= count(S\left(i,j\right)\cong 0)$$

15 $$Vave= \frac{\sum_{i=k-1}^{k+1}\sum_{j=l-1}^{l+1}S(i,j)}{count(S\left(i,j\right)\cong 0)}$$

16 $${S}_{8}\left(i,j\right)=\left\{\begin{array}{c}S\left(i,j\right)= 0 Num<3 \\ S\left(i,j\right)=\frac{\left(S\left(i-1,j\right)+S\left(i+1,j\right)+S\left(i,j\right)-1+S\left(i,j+1\right)\right)}{4} Num > =3 , 1<i<n and 1<j<m\\ \\ S\left(i,j\right)=Vave Num > =3 , i=1 or i=n or j=1 or j=m\end{array}\right.$$

where *1* < *k* and* k* < *n*, *1* < *l* and* l* < *m*, *k* − *1* <  = *i* <  = *k* + *1* and* l* − *1* <  = *j* <  = *l* + *1*.

**Figure 2:**
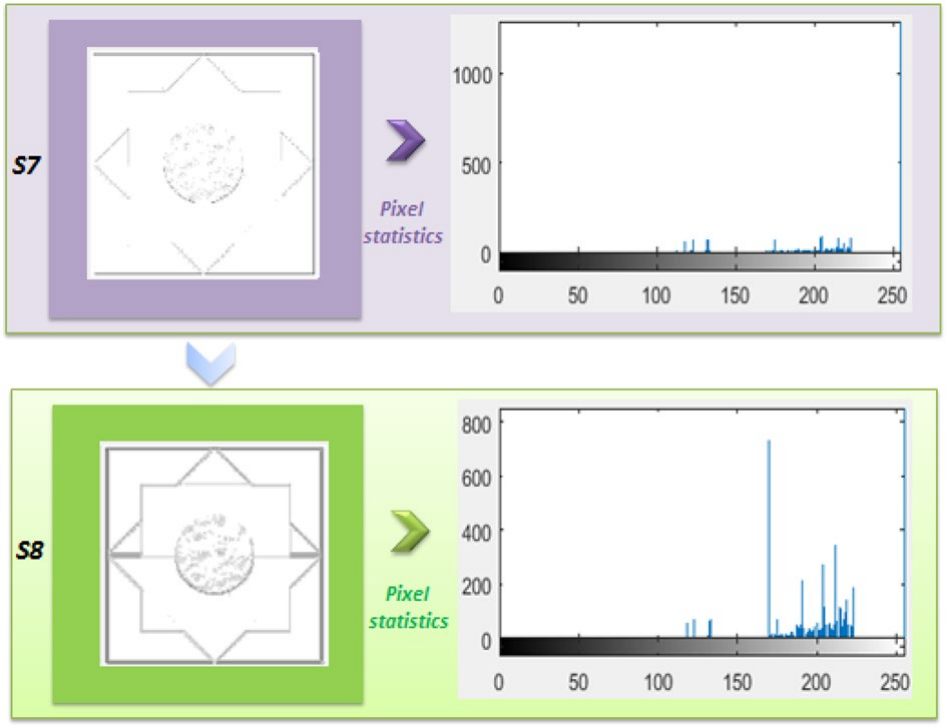
Comparison of image S7, S8 and histogram.

Here is an alternative description: Fig. 2 displays the histogram statistics of the image *S8*, which is generated by expanding the core feature information of image *S7* using a suitable algorithm. As illustrated, the enlarged image has significantly altered the distribution of the original image's feature information, resulting in a decrease in the proportion of pixel values below 30. This reorganization of feature information has shifted the focus of the image to the areas that are easier to identify, improving the image's recognition performance.

## Recognition algorithms

### Flow chart of FCAI algorithm

**Figure 3:**
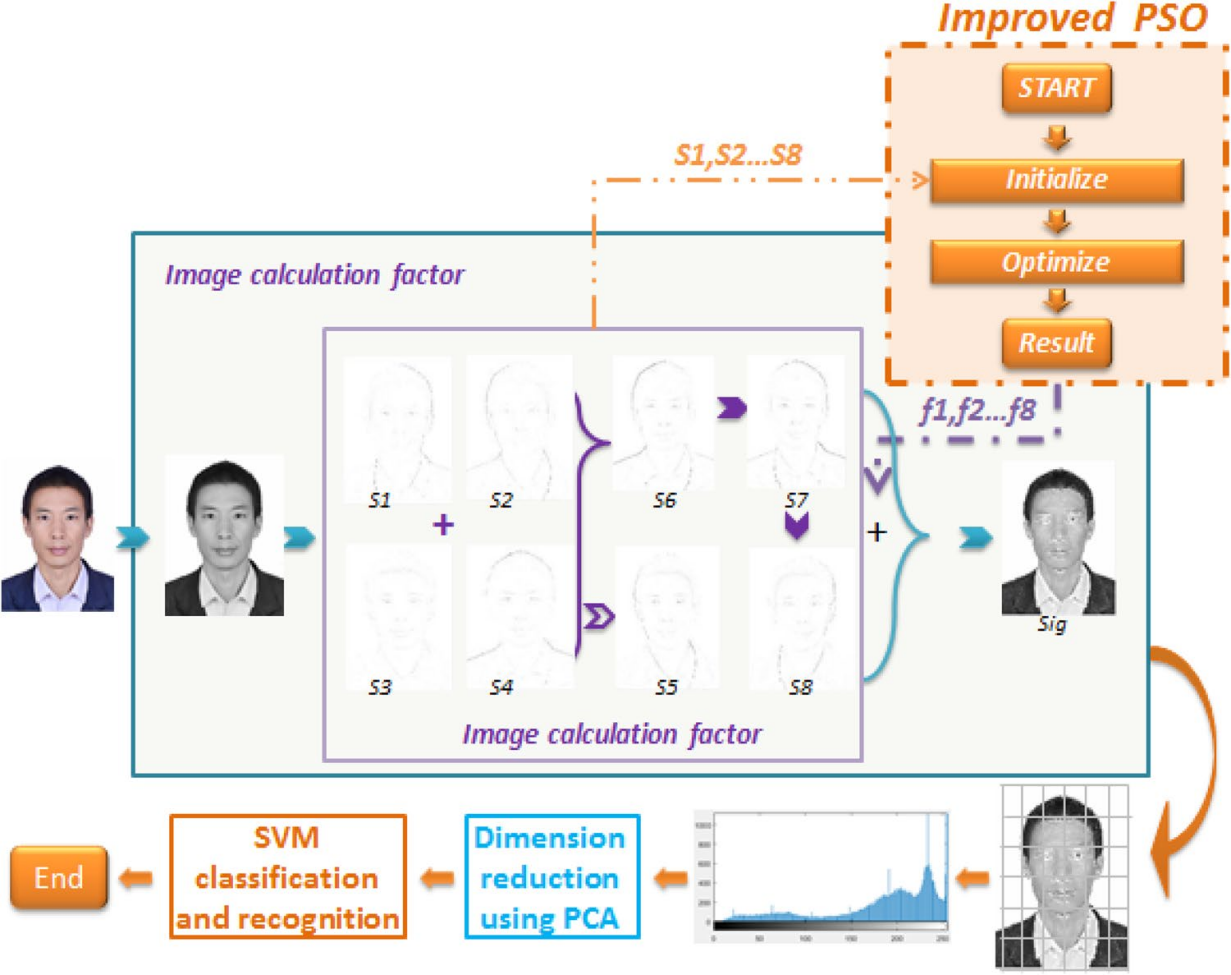
Flow chart of face recognition algorithm.

The flow chart of proposed algorithm is designed as Fig. 3. We have obtained permission from the image owner and are allowed to publish.

Among them, the facial image is the variation of the recommendation algorithm at several important key points.

### Implementation

The implementation process of the FCAI algorithm is as follows: First, the face dataset is converted to gray scale images and imported. Second, factors S1–S8 are calculated using the formulas described in Section “Calculation factors proposed”. Third, an improved PSO algorithm is designed to determine the best feature compensation coefficients for S1–S8. Fourth, the best feature compensation coefficients from step 3 are applied to the image recognition system to generate a feature descriptor image Sig for each original image. Fifth, each Sig image is divided into 36 sub-images using a 6 × 6 template. Sixth, the number of pixels in each of the 36 sub-images is counted. Seventh, the pixel value count results for each of the 36 sub-images are concatenated in a specific order to form a new histogram. Eighth, Principal Component Analysis (PCA) is used to reduce the dimensionality of the original image's histogram, removing non-main feature information and retaining the main feature factors of the image. Ninth, a Support Vector Machine (SVM) algorithm is trained using the PCA results from step 8 to create a training model. Finally, the image recognition system is tested using the trained model.

## Compensation coefficient solving algorithm

When the number of factors for calculating feature compensation is set to 8, with a value range of − 10.00 to 10.00 and an accuracy of 0.01, it becomes impossible to traverse all combinations. In order to address this issue, an improved particle swarm optimization algorithm is proposed in this paper to determine the optimal feature compensation coefficients for the face recognition algorithm.

### Introduction of PSO

The PSO algorithm is a method for optimization that takes inspiration from bird flock behavior and was created by Kennedy and Eberhart^49^. This approach involves a population of m particles that search for the best solution in an n-dimensional search space. Each particle has its own position (P) and velocity (S) and occupies a point in the search space. The position of particle i is represented by an n-dimensional vector Pi = {pi1, pi2 …, Pin}, while its velocity is described by an n-dimensional vector Vi = {vi1, vi2 …, vin}. Unlike physical particles, PSO particles have no volume characteristics. The PSO algorithm updates the positions and velocities of the particles iteratively, based on their own best position and the global best position found by the swarm so far, with the goal of converging to the optimal solution.

### Improved PSO algorithm

#### Flow chart of improved PSO

**Figure 4:**
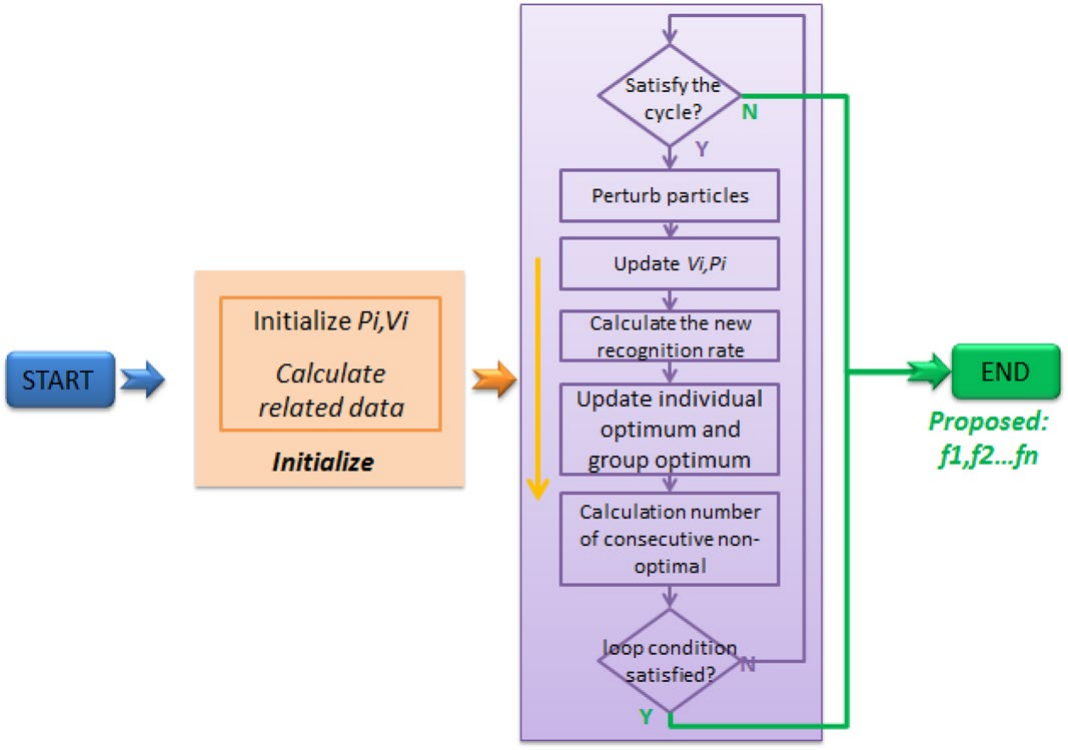
Flowchart of improved PSO algorithm.

The flow chart of improved PSO is designed as Fig. 4.

#### The method of evaluation

The aim is to use the improved PSO algorithm to obtain the best combination of compensation coefficients for the FCAI algorithm. To achieve this, an evaluation algorithm must be developed to assess the strengths and weaknesses of the combination of compensation coefficients. The evaluation algorithm will determine the value of the feature compensation coefficient. The algorithm for evaluating the feature compensation coefficient is designed as follows.

1. Define the algorithm name and the input parameters as 8 feature compensation coefficients *f1*, *f2*, *f3*, *f4*, *f5*, *f6*, *f7*, *f8*.
2. Load the face data set.
3. Extract the feature description of the image which has been compensated by the calculate factor of image using provided combination of the feature compensation coefficients.
4. Decompose the image into 6*6 size small image blocks according to the module.
5. The original image set is decomposed into two parts: training set and test set.
6. Dimension of all images is reduced by serving PCA.
7. Face recognition model is trained by using SVM method on the obtained training set.
8. Test the model using the test set based on the trained face recognition model.
9. Return the face recognition accuracy of the model *fv* and the corresponding coefficient combination.

#### Improved PSO algorithm

The combination of compensation coefficients greatly affects the performance of face recognition systems, making it essential to optimize the combination of compensation coefficients for all feature compensation calculation factors. However, when the number of feature compensation calculation factors is large, it becomes almost impossible to explore all possible combinations. To solve this problem, an improved intelligent algorithm called Particle Swarm Optimization (PSO) algorithm is proposed. Since there are a huge number of combinations when there are eight feature compensation coefficients, we use a combination that is close to the optimal solution instead of the optimal solution.

The improved PSO algorithm considers not only the current position and the optimal solution of the individual and the group, but also the historical best position of the particle, which effectively avoids the algorithm from getting trapped in local optima. To do this, a weight factor is introduced to adjust the influence of the historical best position, and the formula for updating the particle velocity is modified accordingly. This new formula can help the particle swarm to explore the solution space more effectively and accelerate the convergence speed of the algorithm.

In addition to the aforementioned solution, the proposed algorithm also includes a mechanism for adjusting its parameters dynamically during the iteration process. This helps to maintain a balance between exploring and exploiting the solution space and enables the algorithm to adapt to various problem types and complexities.

The algorithm is designed to monitor the number of consecutive iterations that fail to detect any new group optimum solutions during the particle iteration. Based on the maximum number of consecutive undetected new optimum solution iterations set at the start of the algorithm, it will reset the particle position when necessary.

The implementation of the proposed algorithm follows this approach.
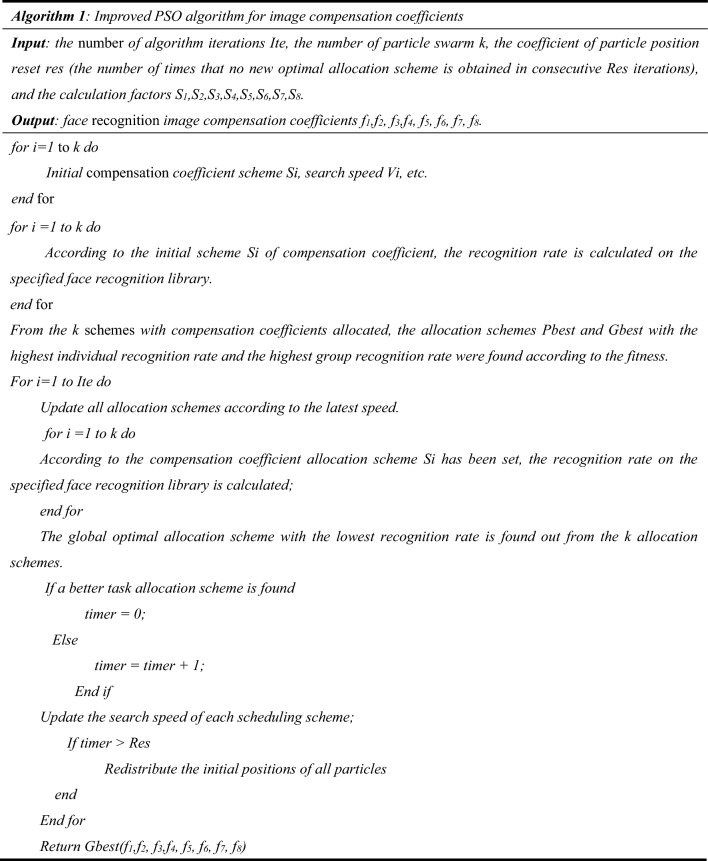


## Experiments

### Experimental environment

In order to verify the recognition effect of the proposed algorithm FCAI, Matlab 2016A is served as the simulation platform for testing. The hardware environment is 8-core 3.4ghz CPU, 24G memory, Windows 7 Professional operating system.

### Correlation data set

To verify the effectiveness of the recommendation algorithm, validations were performed on three separate datasets as follows.

#### ORL face dataset

The ORL face dataset is commonly used to evaluate face recognition algorithms, and it contains images of 40 people. Each individual has 10 images, captured under the same lighting conditions but with varying facial expressions. The database was created by the Livetti Research Laboratory and consists of 4 females and 36 males, with an age range approximately between 18 and 55.

The first 8 images of each person are used as the training set in this study, while the remaining 2 images are used as the test set to assess the accuracy of the algorithm.

#### YALE face dataset

The Yale face dataset is a widely used dataset for face recognition research, containing 15 individuals with 11 images each taken under various conditions such as different lighting, pose, and expression. The database was created by the Department of Computer Science at Yale University and consists of 165 grayscale facial images from 15 participants, including 2 females and 13 males. The age range of the participants is approximately between 18 and 55. The database is intended for research purposes in the fields of facial recognition and computer vision.

The images have a size of 80 × 80 pixels, and the first 8 images of each individual are used as the training set, while the remaining images are used as the test set.

#### MU_PIE face dataset

The MU_PIE face dataset is a dataset created by Carnegie Mellon University for face recognition research. It consists of images of 68 individuals, with each individual having 24 images taken under different lighting conditions, and the images have a size of 64 × 64 pixels. The database was collaboratively created by multiple laboratories within the Computer Science Department of Carnegie Mellon University in the United States. It is a large-scale database of human faces with multiple viewpoints, lighting conditions, and facial expressions, designed for research in face recognition and facial analysis.

In this paper, the first 9 images of each individual are used as the training set, and the remaining images are used as the test set to evaluate the accuracy of the recognition system.

### Experimental results

This paper carries out experiments on three datasets (ORL, YALE, and MU_PIE) to validate the proposed method's effectiveness. Firstly, an improved PSO algorithm is used to obtain the optimal combination of feature compensation coefficients based on a particular dataset (ORL, YALE or MU_PIE), and the performance is validated on that dataset. Secondly, the obtained combination of feature compensation coefficients is applied to the other two datasets, and their recognition rates are compared with multiple popular algorithms to test the scalability of the improved PSO algorithm. To verify the effectiveness of the proposed face recognition algorithm (FCAI), the resulting combination of feature compensation coefficients is used to compare the performance of the LBP algorithm mentioned in^32^, Alg2 algorithm mentioned in^50^, IGP algorithm mentioned in^35^, WLCGP algorithm mentioned in^17^, IGFC algorithm mentioned in^51^ with the proposed algorithm using the parameters related to the experiments as described in the original article.

#### ORL database

The purpose of this study is to test the efficacy of the improved PSO algorithm's recommended combination compensation coefficients on the ORL face dataset. To do this, the study compares randomly generated compensation coefficients with those recommended by the improved PSO algorithm. The recommended compensation coefficients are also compared with those suggested by other popular algorithms. The study analyzes the performance of the improved PSO algorithm fit in three different aspects.

##### Random combination *vs* recommended combination

**Figure 5:**
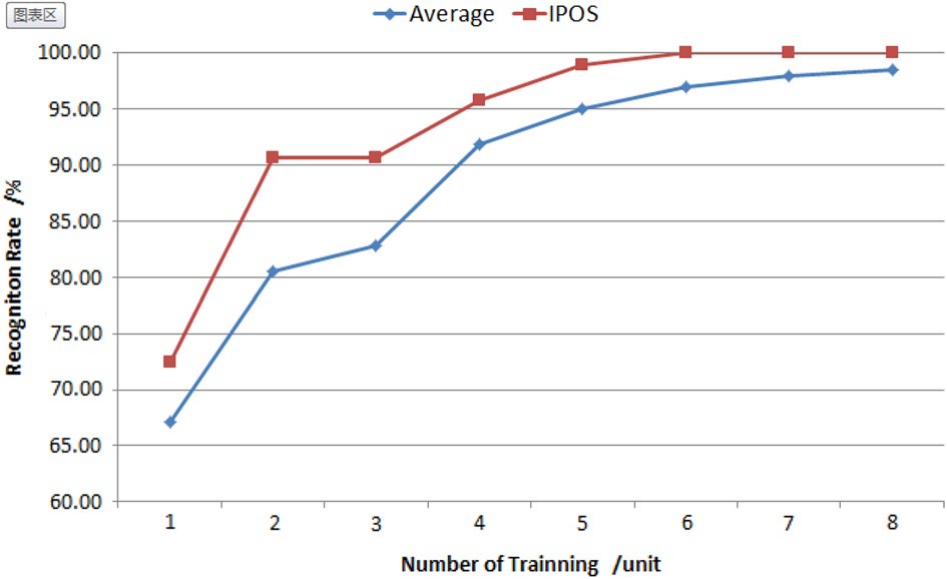
Comparison of recognition rate between combinations of random vs. recommended (%).

The study generates random combinations of 8 features with compensation coefficients between − 10.00 and 10.00, applies them to the ORL dataset for detection, and compares the average results with those obtained using the recommendation algorithm. The results of this comparison are presented in Table 1 and Fig. 5.

**Table 1:** Feature compensation coefficient generated by random method.

Order	*f1*	*f2*	*f3*	*f4*	*f5*	*f6*	*f7*	*f8*
1	2.95	− 0.83	1.65	0.89	− 1.91	3.93	− 3.05	4.73
2	3.58	3.24	0.81	2.95	− 1.03	− 8.12	− 7.00	-2.11
3	2.72	5.41	7.40	0.88	− 2.68	0.51	1.72	3.67
4	8.90	− 3.00	− 4.70	4.42	5.27	0.61	− 4.76	4.08
5	− 5.82	3.24	− 3.64	0.45	2.56	7.22	− 9.11	− 1.15
6	4.19	− 1.68	− 7.62	9.87	5.44	− 0.30	5.10	− 9.61
7	− 5.28	6.84	8.80	− 5.63	8.66	− 2.13	− 5.14	− 3.38
8	− 7.61	6.66	2.91	− 7.88	9.45	3.43	− 1.15	− 1.51
9	2.15	− 4.87	− 0.41	− 7.81	− 6.16	4.83	3.76	− 4.59
10	− 1.00	2.27	2.79	− 8.73	− 7.22	0.40	− 2.82	− 6.06

Figure 5 shows that the improved PSO algorithm can effectively improve the face recognition rate of the system, particularly when the number of training is less than 5.

##### Compare of recognition rates of recommended value *Vs* popular algorithm

**Figure 6:**
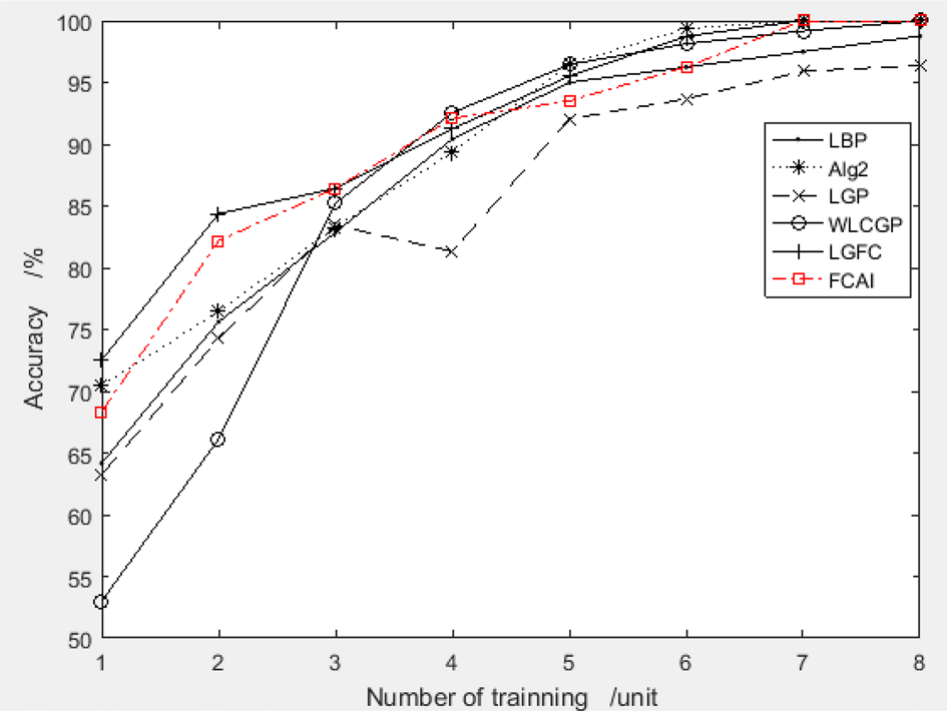
Validation results of recommended feature compensation coefficients on ORL dataset.

The improved PSO algorithm recommends a combination of feature compensation coefficients "9.1495, 4.028, − 0.8900, 3.3404, − 8.2072, − 5.0310, 4.0662, − 9.1498" for the ORL dataset. With 4 training samples, the system achieves a test accuracy of 95.83%, which is highly superior. To evaluate the performance of the proposed combination, the recognition rate is compared with other algorithms for training samples 1–8. The comparison results are presented in Fig. 6.

Figure 6 shows that the FCAI face recognition algorithm, which uses the improved PSO algorithm to suggest the feature compensation coefficient, achieves higher accuracy in face recognition. This indicates that the proposed method can significantly improve image recognition accuracy.

##### Over-fit analysis

**Figure 7:**
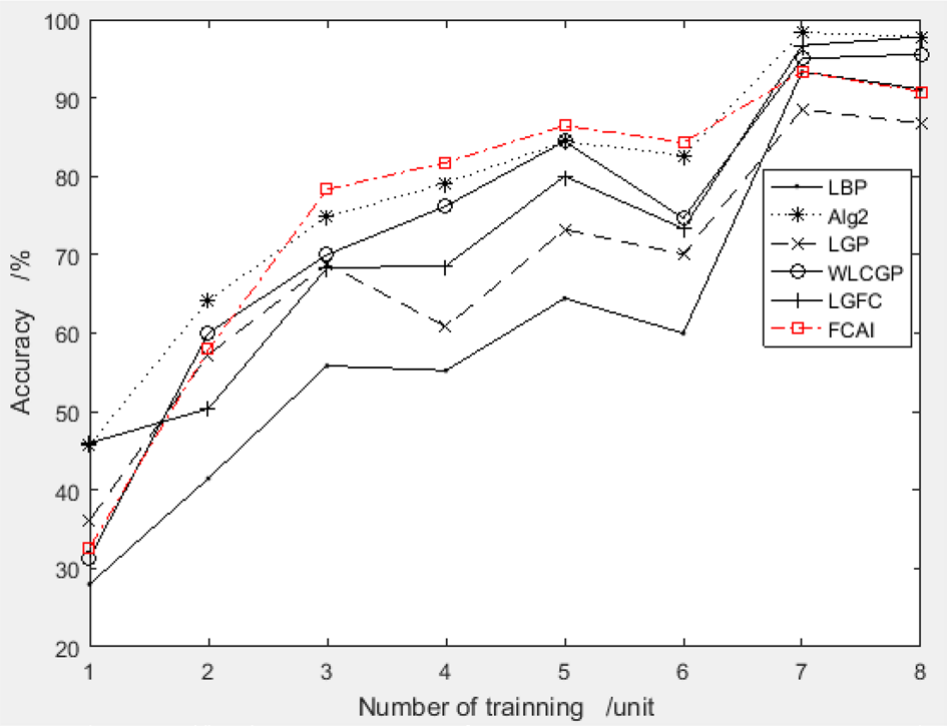
Validation results of recommended feature compensation coefficients on Yale dataset.

**Figure 8:**
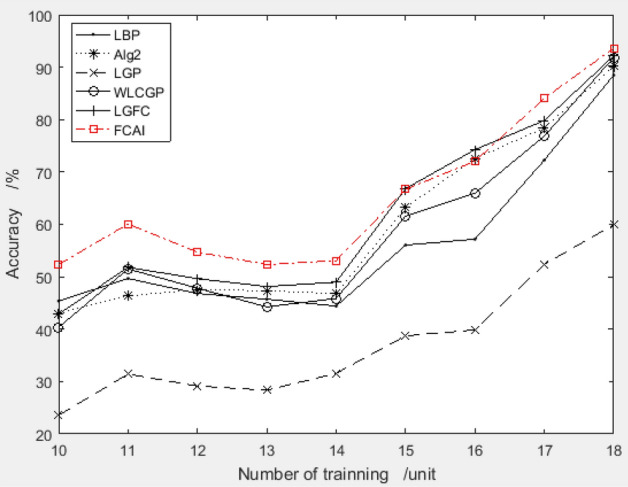
Validation results of recommended feature compensation coefficients on the MU_PIE dataset.

To ensure that the feature compensation coefficient combination obtained from the improved PSO algorithm using the ORL dataset does not have an over-fit problem, the recommended combination is applied to two different datasets, YALE and MU_PIE. The results of the testing are shown in Figs. 7 and 8, respectively. This over-fit analysis helps to confirm the effectiveness of the proposed method across different datasets.

Upon analysis of Figs. 7 and 8, it was found that the recommended feature compensation coefficient combination by the improved PSO algorithm performed exceptionally well on both YALE and MU_PIE datasets. This suggests that the recommended combination is not specific to the training data and can be effectively applied to other datasets, demonstrating its generalizability and applicability.

#### YALE database

To evaluate the performance of the face recognition system using the feature compensation coefficient combination recommended by the improved PSO algorithm on YALE dataset, we conducted a detailed experimental verification similar to the one conducted in Section 5.2.1. The improved PSO algorithm suggested a combination of "− 2.5601, 2.0008, 1.0244, 4.4897, 3.4257, 0.5377, − 3.7378, − 0.7328" for feature compensation.

##### Recognition rate on YALE

We then tested the recognition rate of the face recognition system on YALE dataset using this combination. The recognition rate was compared with other methods, and the results are presented in detail in Fig. 6. We conducted experiments using 1 to 8 training samples for each person.

**Figure 9:**
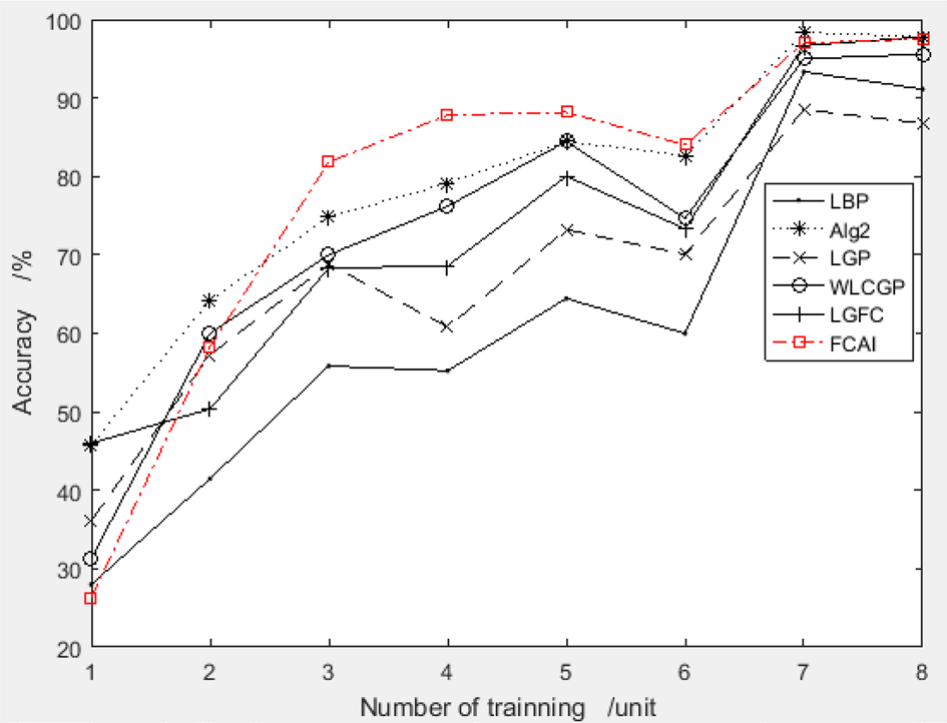
Validation results of recommended feature compensation coefficients on Yale dataset.

It can be seen from Fig. 9 that the accuracy of the FCAI faces recognition algorithm with the recommended feature compensation coefficients using the proposed improved PSO algorithm still performs well.

##### The application effect on ORL, MU_PIE face dataset

**Figure 10:**
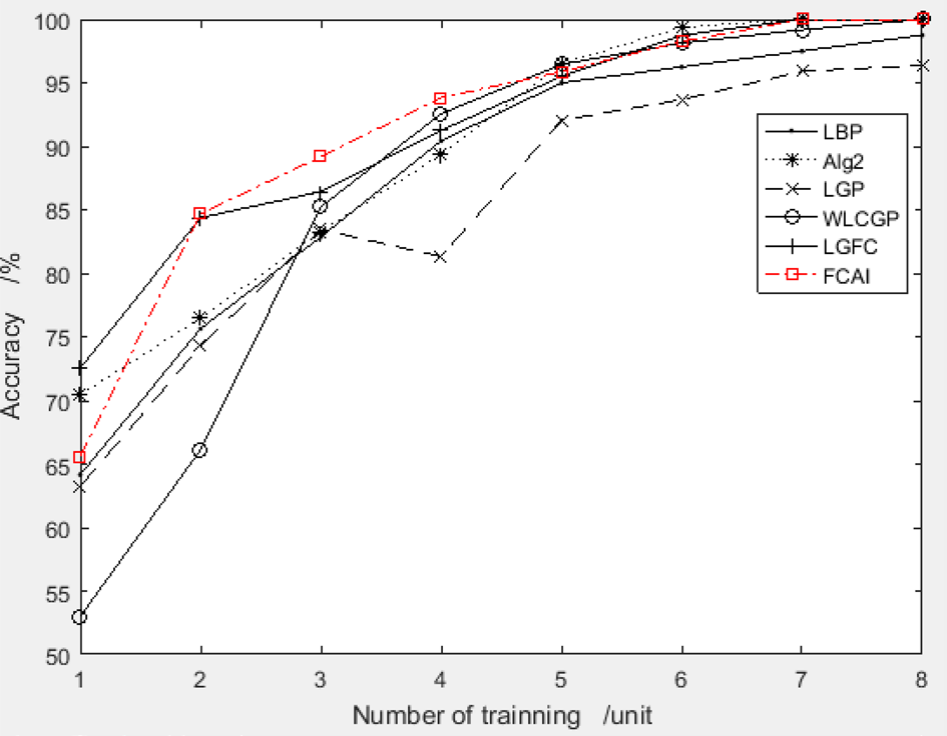
Validation results of recommended feature compensation coefficients on the ORL dataset.

**Figure 11:**
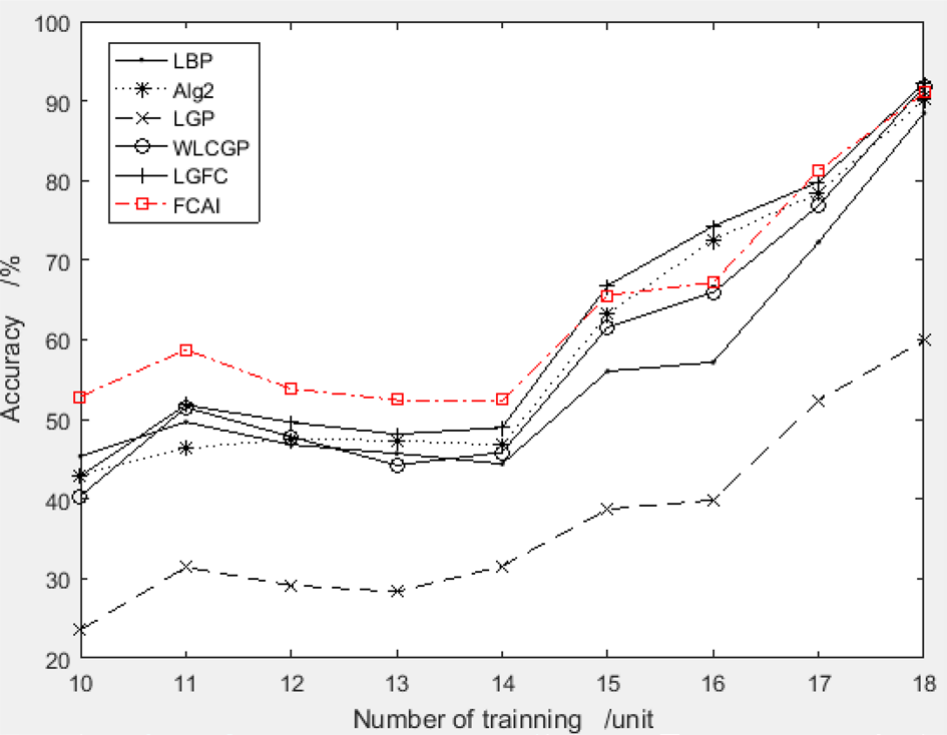
Validation results of recommended feature compensation coefficients on the MU_PIE dataset.

The results of the above figures, Figs. 10 and 11, show that the feature compensation coefficient generated based on YALE dataset still has a wonderful performance on ORL dataset and MU_PIE dataset, especially on MU_PIE face dataset, where the performance of the feature compensation coefficients rides high and the realizations are exceptionally good.

#### MU_PIE database

A detailed experimental verification is conducted in this paper to test the recognition rate of the system using the feature compensation coefficient group recommended by the improved PSO algorithm on the MU_PIE dataset. The algorithm suggests the combination of "3.3004, 2.2134, 3.7822, 6.1612, 2.2649, − 4.1176, − 0.8248, 0.2291" for feature compensation. The results demonstrate the effectiveness of the proposed algorithm in improving the recognition rate of the face recognition system.

##### Recognition rate on MU_PIE

**Figure 12:**
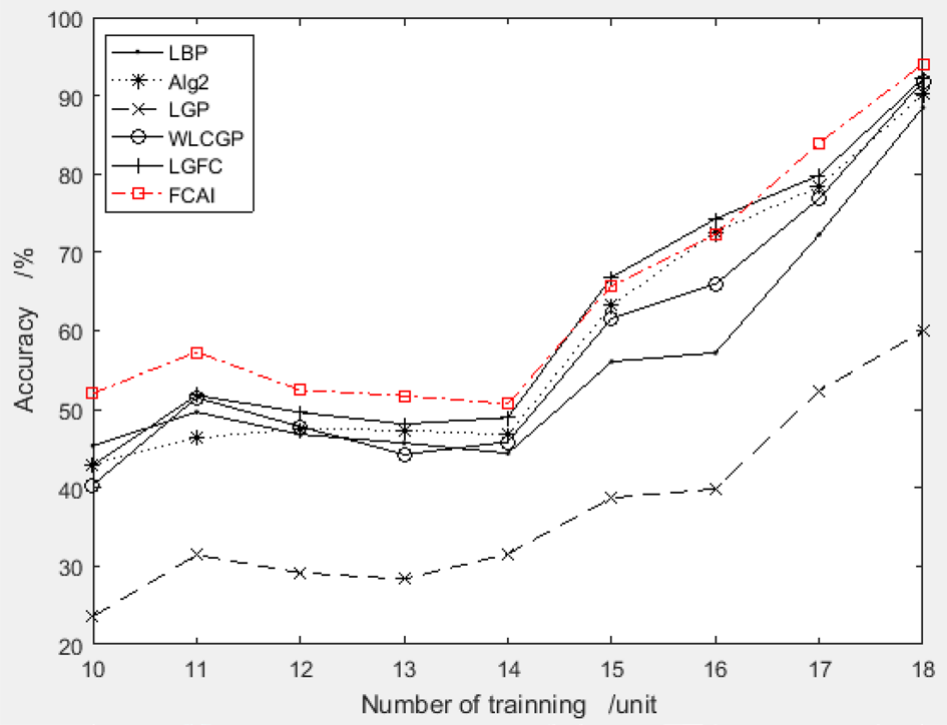
Validation results of recommended feature compensation coefficients on MU_PIE dataset.

The paper conducted a detailed experiment to verify the performance of the feature compensation coefficient group recommended by the improved PSO algorithm on the MU_PIE dataset. The system used the combination "3.3004, 2.2134, 3.7822, 6.1612, 2.2649, − 4.1176, − 0.8248, 0.2291" for feature compensation, and the training samples were selected from the first 10 to 18 images of each person. The recognition rate was compared with other algorithms, and the results are presented in Fig. 12.

An outstanding result is obtained from the above figure, which shows that the face recognition algorithm utilizing the feature compensation coefficient combination recommended by the improved PSO algorithm has superior accuracy compared to other widely used face recognition algorithms.

##### The application effect on ORL and YALE face datasets

**Figure 13:**
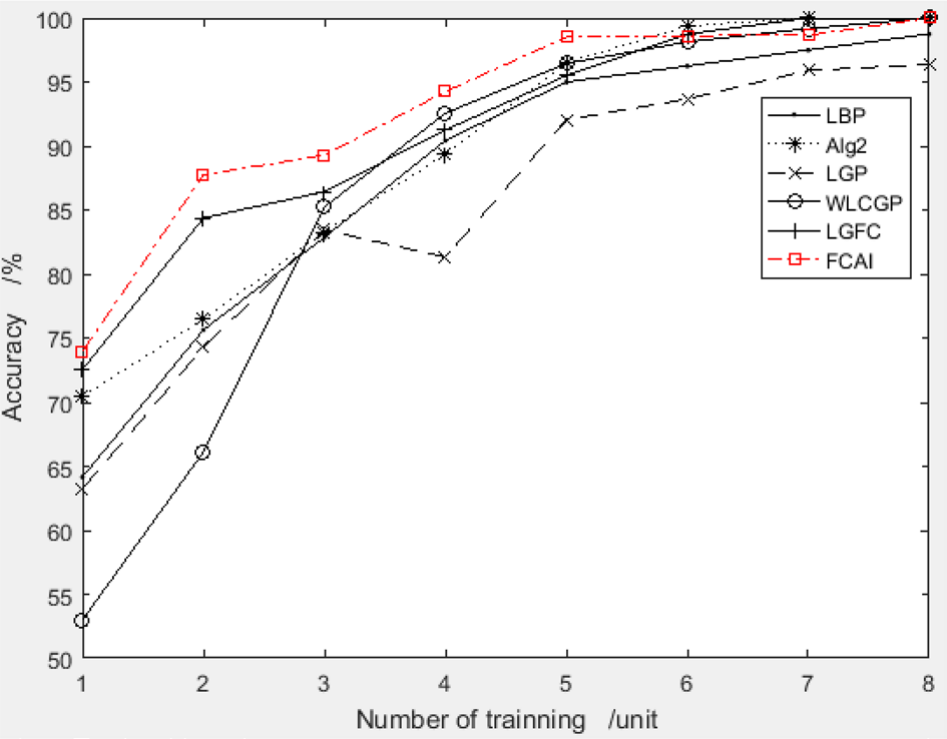
Validation results of recommended feature compensation coefficients on the ORL dataset.

**Figure 14:**
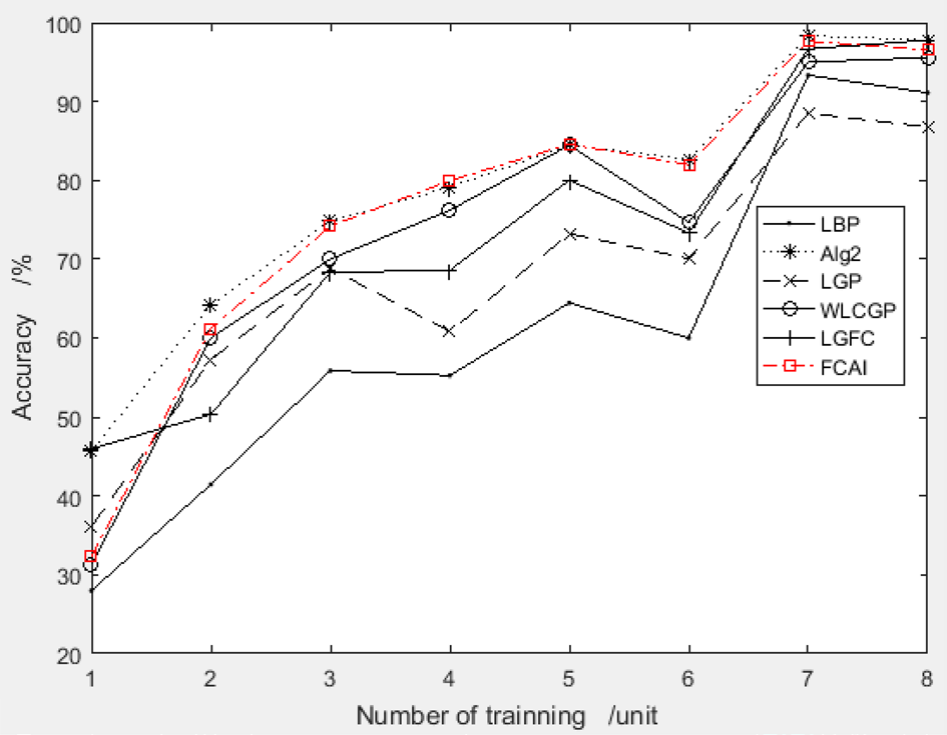
Validation results of recommended feature compensation coefficients on the YALE dataset.

Based on the examination of Figs. 13 and 14, it is evident that the feature compensation coefficient created using the MU_PIE dataset does not perform the best on the ORL and YALE datasets. However, the results are generally satisfactory, with the coefficient combination demonstrating impressive performance on the YALE dataset.

In conclusion, the proposed improved PSO algorithm is effective in optimizing the accuracy of the face recognition system by incorporating the compensation strategy. The experimental results indicate that the feature compensation coefficients generated by the algorithm do not suffer from significant over-fitting and have practical value for promoting and using in real-world applications.

## Conclusions

A novel face recognition algorithm based on fusion of image feature compensation and improved PSO (FCAI) is proposed for improving the face recognition accuracy, and a method that extracts the image feature description by using the computational factors to compensate the original image features is adopted in this paper. Due to the values of feature compensation coefficients could directly affect the recognition rate of the system, a modified PSO algorithm is proposed to solve the optimal combination of compensation coefficients for multiple computation factors. The experimental results that have been finished on the simulation platform show that when the improved PSO algorithm is applied to the proposed FCAI algorithm, the recognition rate of the proposed algorithm can be significantly improved, and there is no over-fit problem.

## Limitations of the study

This study did not delve deeper into the proposed computational factors and failed to uncover which computational factor contributes more significantly to the facial recognition system. In future research, a more in-depth investigation will be conducted.

## Data Availability

The data that support the findings of this study are available from the corresponding author upon reasonable request.
